# Assessment of Interleukin-15 (IL-15) Concentration in Children with Cystic Kidney Disease

**DOI:** 10.3390/biomedicines14091982

**Published:** 2026-09-02

**Authors:** Anna Bogdał, Aleksandra Hop, Elżbieta Świętochowska, Artur Janek, Tomasz Morelewski, Andrzej Badeński, Katarzyna Otrębska, Maria Szczepańska

**Affiliations:** 1 Department of Pediatrics, District Hospital in Zawiercie, Miodowa Street 14, 42-400 Zawiercie, Poland; ania.bog94@wp.pl; 2 Chair and Clinical Department of Pediatrics, Hospital No. 2 in Bytom, ul. Stefana Batorego 15, 41-902 Bytom, Poland; 3 Department of Medical and Molecular Biology, Faculty of Medical Sciences in Zabrze, Medical University of Silesia, 40-055 Katowice, Poland; elaswieta@interia.pl; 4 Department of Pediatrics, Faculty of Medical Sciences in Zabrze, Medical University of Silesia in Katowice, ul. 3 Maja 13/15, 41-800 Zabrze, Poland; a.janek97@gmail.com (A.J.); tmorelew.sky@gmail.com (T.M.); mszczepanska@sum.edu.pl (M.S.); 5 Praski Hospital of the Transfiguration of the Lord, al. Solidarności 67, 03-401 Warsaw, Poland; kat.otrebska@gmail.com

**Keywords:** interleukin-15, cystic kidney disease, pediatric nephrology

## Abstract

Cystic kidney disease is characterized by progressive structural remodeling of renal parenchyma and altered cellular signaling. Inflammatory mediators may contribute to disease biology; however, data on interleukin-15 (IL-15) in pediatric cystic kidney disorders remain limited. The aim of this study was to assess serum and urinary IL-15 concentrations in children with cystic kidney disease and to evaluate their association with renal function. **Methods**: This study included 47 children with cystic kidney disease and 41 controls without renal cystic disease or other systemic, inflammatory, or immunological disorders. Serum and urinary IL-15 concentrations were measured using an enzyme-linked immunosorbent assay (ELISA). Renal function was assessed using the estimated glomerular filtration rate (eGFR). Statistical analyses included non-parametric comparisons, logistic regression, and receiver operating characteristic (ROC) curve analysis. **Results**: Children with cystic kidney disease demonstrated significantly elevated serum and urinary IL-15 concentrations compared with controls. IL-15 levels were not associated with eGFR and did not differ between cystic disease subtypes. In logistic regression analysis, IL-15 remained significantly associated with disease status. ROC analysis suggested high diagnostic ability of serum and urinary IL-15. **Conclusions**: IL-15 concentrations are increased in children with cystic kidney disease independently of current renal function. These findings suggest that IL-15 may reflect early inflammatory or epithelial signaling rather than established functional impairment.

## 1. Introduction

### 1.1. Renal Cystic Disease

Renal cystic disease is a heterogeneous group of disorders that differ in etiology and clinical presentation [1]. Renal cysts are fluid-filled structural modifications of the kidney that may develop in different segments of the nephron and whose evolution and progression are influenced by cellular and molecular processes [1,2]. Single renal cysts have limited clinical significance, but the presence of multiple cysts may lead to progressive loss of kidney function [1]. Within genetic disorders, autosomal dominant polycystic kidney disease (ADPKD) and autosomal recessive polycystic kidney disease (ARPKD) represent the main genetic forms [1]. ADPKD is caused by pathogenic variants in *PKD1* or *PKD2* genes, encoding polycystin 1 and polycystin 2 [1,3,4,5]. ADPKD occurs with a frequency of 1 in 400 to 1 in 1000 individuals and is characterized by bilateral, progressive cyst formation [3,4,6]. ARPKD occurs with a frequency of 1 in 20,000 live births and is caused by mutation in *PKHD1* encoding fibrocystin [3,4]. Other genetically determined cystic kidney diseases include autosomal dominant tubulointerstitial kidney disease (ADTKD) [1]. The non-genetically determined group includes multicystic dysplastic kidney (MCDK), medullary sponge kidney (MSK) and isolated renal cysts [1]. MCDK is characterized by renal enlargement with cysts of various sizes and the absence of normal renal parenchyma [7]. MCDK is most commonly unilateral and bilateral involvement is associated with an unfavorable prognosis and a high rate of perinatal mortality [7,8]. MSK is a developmental disorder in which cysts arise within the renal medulla, most commonly in a bilateral distribution, accompanied by dilatation of the collecting ducts [1]. Isolated renal cysts are most often identified incidentally as single cortical lesions and do not impair renal function [7]. Differentiating these entities at the molecular and clinical levels may improve diagnostic accuracy and support the development of targeted therapeutic approaches [9].

### 1.2. Interleukin-15

Interleukin-15 (IL-15) is a pro-inflammatory cytokine that promotes T-cell proliferation, enhances the cytotoxic activity of T cells and natural killer cells, and protects T cells and neutrophils from apoptosis [10]. IL-15 can stimulate the production of other pro-inflammatory cytokines, thereby contributing to the amplification of immune responses [10]. IL-15 is a glycoprotein with a molecular mass of 14–15 kDa [11]. IL-15 belongs to the IL-2 family of cytokines, which are characterized by their dependence on the gamma chain receptor for signaling [11]. IL-15 is expressed by immune and non-immune cell populations, including epithelial cells [11,12]. Expression of the *IL-15* gene has been identified in numerous tissues, including the kidney [10]. IL-15 is produced by monocytes, macrophages, and dendritic cells, and expression can be induced during bacterial and viral infections through innate immune signals [13]. Within the kidney, both IL-15 and its receptor are expressed and have an important role in promoting survival signaling in renal epithelial cells [11] Human and murine tubular epithelial cells express IL-15 and its receptor, contributing to local epithelial pro-survival signaling [14]. Experimental nephritis studies suggest that tubular epithelial IL-15 expression contributes to epithelial cell preservation and may reduce susceptibility to immune-mediated renal injury [12]. IL-15 has been reported to limit the epithelial–mesenchymal transition in tubular epithelial cells through inhibition of TGF-β1 expression and signaling, as well as to reduce renal matrix deposition by affecting collagen synthesis in myofibroblasts and decreasing macrophage recruitment [11,15]. Beyond tubular cells, IL-15 activates STAT5 in podocytes, where it enhances podocyte survival pathways associated with autophagy regulation in focal segmental glomerulosclerosis [11,14]. Exogenous IL-15 or IL-15/IL-15Rα complexes reduce proteinuria and preserve podocyte ultrastructure, which is a protective role of this cytokine in glomerular disease [10,14]. IL-15–STAT5 signaling is an important survival pathway in the glomerular epithelium [11,14]. Understanding how IL-15 signaling is modulated in different renal diseases is essential for evaluating its potential as a diagnostic and therapeutic target in cystic disease [9,11].

The aim of this study was to assess serum and urinary IL-15 levels in children with renal cysts and to determine whether IL-15 could serve as a potential biomarker in cystic kidney disease.

## 2. Materials and Methods

### 2.1. Study Groups

Patients representing the study group were children aged below 18 years (n = 47) with renal cystic disease. They were under the care of the Department of Pediatric Nephrology with the Subdivision of Dialysis and the Pediatric Nephrology Outpatient Clinic at the Clinical Hospital No. 1 in Zabrze, Medical University of Silesia in Katowice, between 2016 and 2023. The study group consisted of 25 girls (53.2%) and 22 boys (46.8%). Inclusion criteria for the study group comprised age below 18 years and the presence of at least one renal cyst detected on ultrasonographic examination. Exclusion criteria included the absence of informed consent to participate in the study and acute infections at the time of clinical evaluation or biological sample collection. Among patients with renal cystic disease, 23 children (48.9%) were diagnosed with ADPKD, 10 patients (21.3%) presented with simple renal cysts, and 14 patients (29.8%) were classified as having another cystic kidney disease. The diagnosis of renal cystic disease was established based on ultrasonographic findings and a review of medical records. The diagnosis of ADPKD was based on a combination of clinical and radiological criteria and genetic testing, where available.

The control group consisted of 41 children under the age of 18 without cystic kidney lesions (17 girls, 41.5%, and 24 boys, 58.5%), who were evaluated at the same center during the same period due to non-nephrological conditions. These included patients hospitalized for the diagnostic evaluation of functional nocturnal enuresis, which resolved following psychotherapy, and patients admitted for surgical release of the lingual frenulum. None of the control participants had any other somatic, inflammatory or immunological disorders.

Overall, 88 patients were included in the study, comprising the study group (n = 47) and the control group (n = 41).

This research project was approved by the Bioethics Committee of the Silesian Medical Chamber, No. 37/2021 (27 September 2021). Written informed consent was obtained from the caregivers of all children in both the studied and control groups and, in the case of participants older than 16 years, also from the child.

### 2.2. Laboratory Tests

Routine hematological, biochemical, and urinary laboratory tests were performed in all participants as part of standard clinical care. Blood analyses included a complete blood count (hemoglobin, red blood cells, white blood cells, platelets) and basic biochemical parameters (serum creatinine, serum urea, serum uric acid, electrolytes, total protein, albumin, C-reactive protein, alanine aminotransferase, aspartate aminotransferase, blood glucose).

Urine testing comprised standard urinalysis and a quantitative assessment of selected urinary indices (urine specific gravity, proteinuria, leukocyturia, erythrocyturia, urinary creatinine, urinary urea, urinary uric acid, urinary calcium, urinary phosphate, urinary sodium, urinary potassium, urinary albumin). Renal function was evaluated using the serum creatinine concentration and estimated glomerular filtration rate (eGFR), calculated according to the Schwartz formula [16].

### 2.3. IL-15 Concentration

Serum and urine concentrations of IL-15 were determined using an enzyme-linked immunosorbent assay (ELISA) with the commercially available Human IL-15 Immunoassay kit (R&D Systems, Minneapolis, MN, USA; Cat. No. D1500). Each biological sample was analyzed in triplicate, and the reported values represent the arithmetic mean of the measurements obtained. All analyses were carried out strictly according to the manufacturer’s protocol provided with the assay kit. Absorbance was measured at 450 nm with correction at 570 nm using a SYNERGY/H1 microplate reader (BioTek, Santa Clara, CA, USA). Data processing and calculation of the results were performed using Gen5 software version 3.05 (BioTek, Santa Clara, CA, USA). The assay sensitivity was 0.2 pg/mL, and the intra-assay coefficient of variation, reflecting method precision, was 5.1%.

### 2.4. Anthropometric and Clinical Measurements

Anthropometric measurements, including body weight (in kilograms) and body height (in centimeters), were collected for all participants and reported to two decimal places. For each individual, body mass index (BMI) was calculated using the formula weight/height^2^ (kg/m^2^), and blood pressure was measured as part of routine assessment. Age- and sex-specific percentile charts from the OLA and OLAF studies for Polish children [17,18] were used to evaluate the above-mentioned parameters. Additionally, standard deviation scores (SDSs) were calculated to facilitate comparisons between the study groups. No missing data were identified in the analyzed population.

### 2.5. Statistical Analysis

Statistical analysis was conducted using RStudio software (version 2026.08.2) with the R programming language. Descriptive statistics were presented as means with standard deviations or as medians with interquartile ranges, depending on the data distribution. The Shapiro–Wilk test was used to assess the normality of the data distribution.

Comparisons between the study and control groups were performed using Student’s t-test or non-parametric tests, as appropriate. Correlations between IL-15 concentrations and selected clinical parameters were evaluated using Spearman’s rank correlation coefficient. Logistic regression models were constructed to assess the association between IL-15 levels and the presence of renal cysts and ADPKD. Receiver operating characteristic (ROC) curve analysis was used to evaluate the diagnostic performance of serum and urinary IL-15. Statistical significance was set at *p* < 0.05.

Data are presented as mean ± standard deviation (SD) for normally distributed variables and as median (Q1–Q3) for non-normally distributed variables. Variables that demonstrated statistical significance in the univariable analysis were entered into the multivariable logistic regression model. The diagnostic performance of biomarkers was interpreted according to commonly accepted AUC thresholds. Due to incomplete laboratory records and occasional sample contamination, the number of valid observations differed between selected variables.

Cohen’s d was used as the effect size, and statistical power was calculated using the pwr.t.test function in the pwr package in R (version 4.6.1.).

## 3. Results

### 3.1. Characteristics of the Study Population

No significant difference in sex and age distribution was observed between the groups. Overall renal function was preserved in the study group, with a mean serum creatinine concentration of 48.4 ± 12.6 µmol/L and a mean estimated glomerular filtration rate (eGFR) of 116.7 ± 23.1 mL/min/1.73 m^2^. Descriptive statistics for anthropometric, clinical, and laboratory variables are presented in Table 1.

### 3.2. Serum and Urinary IL-15 Concentrations

**Figure 1:**
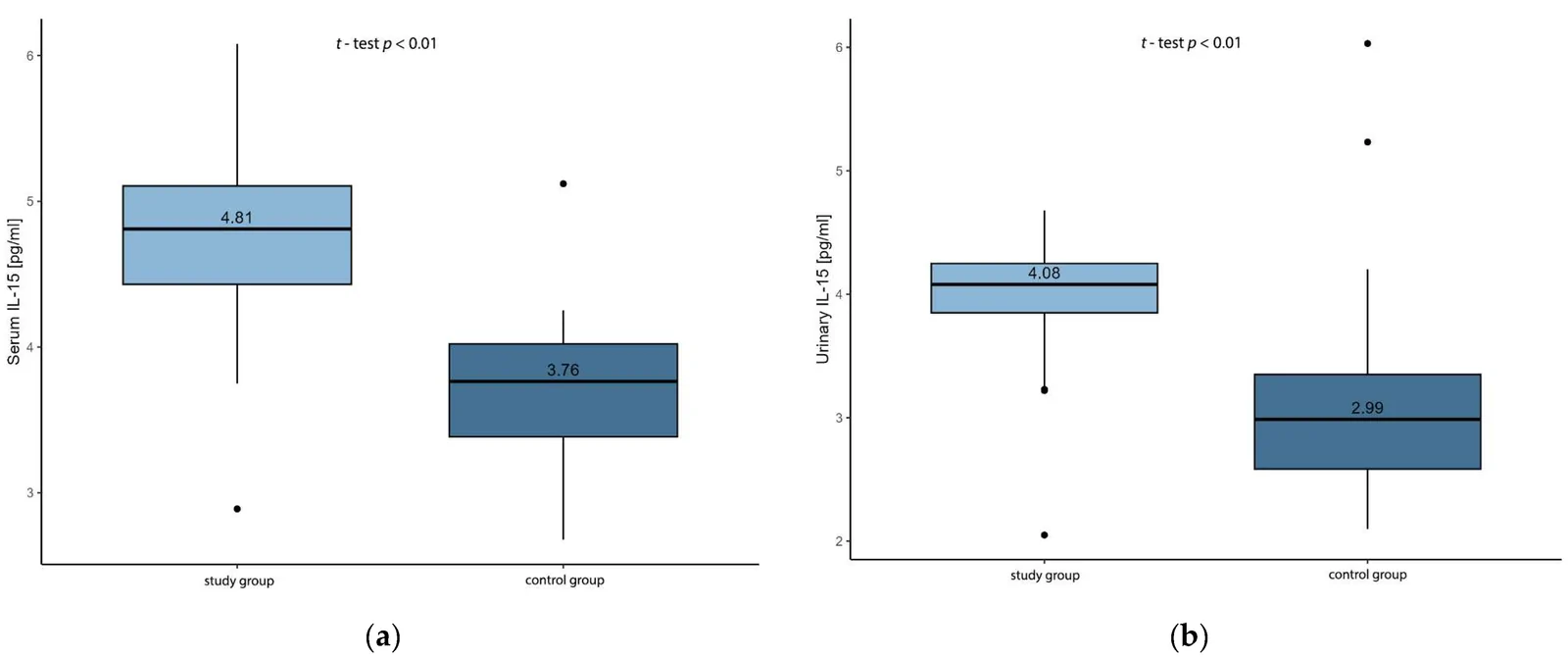
Comparison of IL-15 concentrations between children with renal cystic disease and controls: (**a**) serum; (**b**) urinary. Data are presented as box-and-whisker plots showing the median, interquartile range, and minimum–maximum values. Statistical significance was assessed using the Mann–Whitney U test, as IL-15 distributions deviated from normality.

Serum IL-15 concentrations were significantly higher in patients with renal cystic disease compared with the control group (*p* < 0.001). Similarly, urinary IL-15 concentrations were significantly increased in the study group relative to controls (*p* < 0.001). Comparisons of serum and urinary IL-15 concentrations between the study and control groups are illustrated in Figure 1a,b.

When patients with renal cystic disease were stratified according to cyst subtype (ADPKD, simple renal cysts, and other cystic kidney diseases), no significant differences in serum or urinary IL-15 concentrations were observed between the subgroups. In univariable logistic regression analyses assessing factors associated with the presence of ADPKD, serum and urinary IL-15 concentrations were not identified as significant predictors (Table 2).

Univariable logistic regression analyses demonstrated that higher serum and urinary IL-15 concentrations were associated with a significantly increased likelihood of the presence of renal cysts. Both serum IL-15 and urinary IL-15 emerged as significant predictors in univariable models (Table 3).

### 3.3. Correlations Between IL-15 and Clinical and Biochemical Parameters

Spearman correlation analysis showed no significant association between serum IL-15 concentration and CRP (R = −0.040, *p* = 0.805) or eGFR (R = 0.115, *p* = 0.440). Similarly, urinary IL-15 concentration did not correlate significantly with CRP (R = 0.002, *p* = 0.989) or eGFR (R = −0.021, *p* = 0.889). A significant positive correlation was observed between serum and urinary IL-15 concentrations (R = 0.634, *p* < 0.001). The results of the correlation analysis between serum and urinary IL-15 concentrations and biochemical variables are presented in Table 4.

### 3.4. Diagnostic Performance of IL-15

**Figure 2:**
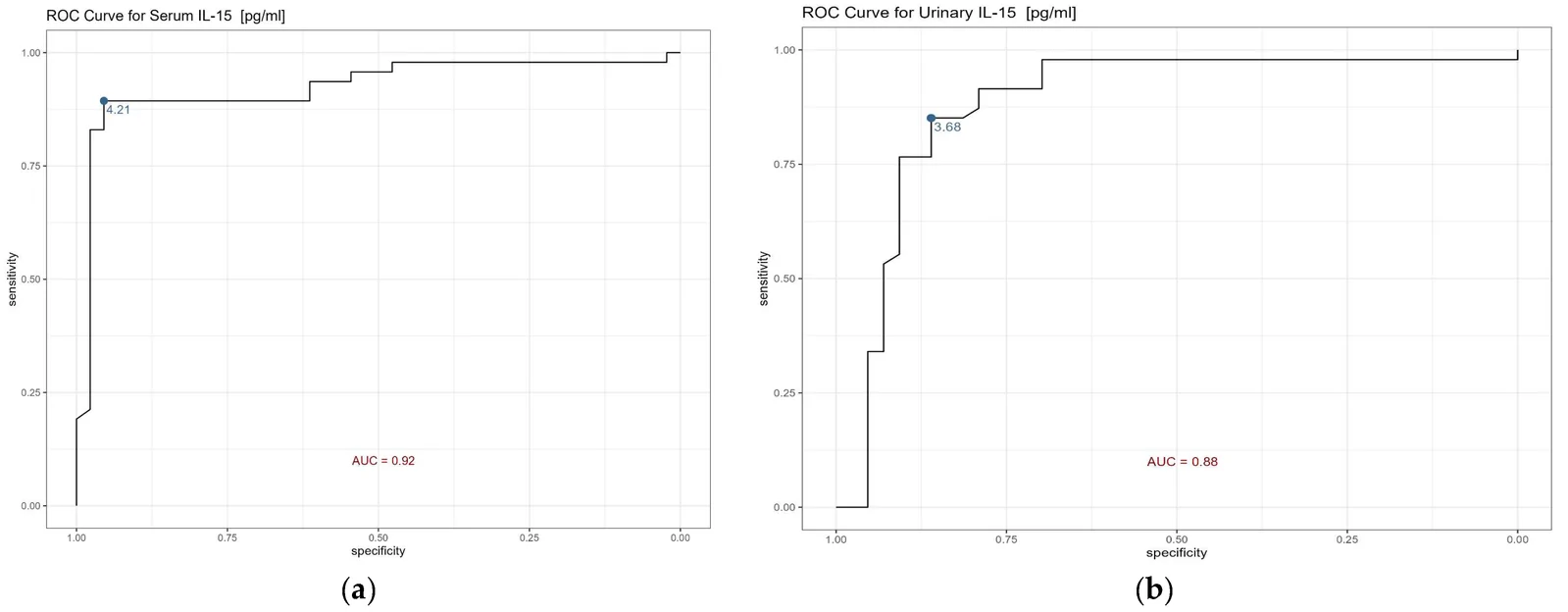
ROC curve for IL-15 concentration: (**a**) serum; (**b**) urine.

ROC curve analysis demonstrated high diagnostic ability for serum IL-15 (AUC = 0.92) and urinary IL-15 (AUC = 0.88) for differentiating children with renal cystic disease from controls. However, these findings should be interpreted cautiously due to the relatively small sample size and the lack of model validation. ROC curves for serum and urinary IL-15 are shown in Figure 2a,b, and the corresponding diagnostic parameters are summarized in Table 5.

## 4. Discussion

Cystic kidney disease is characterized by epithelial proliferation, altered cellular signaling, and progressive structural remodeling [5,9,19]. Adult studies have demonstrated metabolic reprogramming of cystic epithelial cells, including increased glycolysis, mTOR activation, and suppression of mitochondrial oxidative phosphorylation [5,19]. Inflammatory signaling pathways involving IL-1β, IL-6, TNF-α, and IFN-γ are upregulated in advanced stages of disease [19,20]. Most of these data arise from adult cohorts and represent later stages of disease [5,19,20]. Pediatric biomarker studies remain limited, and blood or urinary markers predicting disease progression in children have not yet been validated [21]. In our study, we demonstrated significantly elevated serum and urinary IL-15 concentrations in children with cystic kidney disease. IL-15 levels were not associated with eGFR and did not differ between cystic disease subtypes like simple renal cysts, ADPKD and others renal cysts. These findings suggest that IL-15 elevation is not directly associated with renal function in children with cystic kidney disease. A large adult ADPKD cohort study confirmed systemic cytokine activation but demonstrated diverse correlation with renal function. Arjune et al., in a cohort of 233 adults with ADPKD, reported significantly elevated IL-6, IL-8, MCP-1, TNF-α, and IFN-γ compared with controls; only TNF-α showed a significant inverse correlation with eGFR, while other cytokines did not [20]. Ene et al. demonstrated that dysregulation of the IL-12 cytokine family in ADPKD correlated with decreasing eGFR and larger kidney volume; IL-35 showed inverse associations [22]. These findings suggest that cytokine activation in ADPKD may occur independently of a measurable decline in renal function. Pediatric data on IL-15 remain limited but highly relevant. Badeński et al. demonstrated elevated IL-15 concentrations in both serum and urine in children with idiopathic nephrotic syndrome [10]. No correlations were observed between IL-15 and eGFR, CRP, proteinuria, or total protein levels [10]. Serum and urinary IL-15 concentrations did not correlate with each other, and IL-15 levels were not influenced by immunosuppressive therapy [10]. These pediatric observations are consistent with the findings of our cohort and suggest that IL-15 upregulation in children with kidney disease may occur independently of a measurable functional impairment. The biological role of IL-15 in renal tissue provides additional information. IL-15 is expressed by renal tubular epithelial cells and signals through the IL-15 receptor complex, activating JAK/STAT pathways [4,11,14]. Shinozaki et al. demonstrated that IL-15 deficiency in nephritis models resulted in increased tubular apoptosis and worsened renal injury; recombinant IL-15 reduced apoptosis and improved functional parameters [12]. Niasse et al. showed that IL-15-mediated activation of STAT5 in podocytes attenuated structural damage and reduced albuminuria in experimental glomerular injury [23]. IL-15 supports epithelial stability and inhibits the epithelial-to-mesenchymal transition via JAK/STAT activation [14]. These findings support a cytoprotective and homeostatic role of IL-15 within the renal epithelium. IL-15 may influence renal injury in autoimmune conditions. Zhang et al. showed elevated serum IL-15 levels in lupus nephritis patients and demonstrated expansion and renal infiltration of CD4^+^CD28^−^ T cells [13]. In that cohort, increased CD4^+^CD28^−^ T-cell infiltration was associated with a reduced eGFR and greater proteinuria and glomerulosclerosis [13]. IL-15 stimulation enhanced cytotoxic molecule expression and endothelial cell apoptosis in vitro [13]. The available data demonstrate that IL-15 contributes to cytotoxic immune mechanisms in autoimmune nephritis, whereas in cystic kidney disease, increased concentrations are observed despite preserved renal function. In children with cystic kidney disease, renal function remains preserved despite ongoing structural changes [21]. Adult studies suggest that inflammatory activation may precede a measurable decline in the eGFR [20,22]. Elevated IL-15 in our cohort may represent an early biological signal not yet translated into functional impairment. The relationship between IL-15 and structural disease parameters in pediatric cystic kidney disease remains to be clarified, as such measures were not assessed in our study and are rarely reported in pediatric IL-15 studies [10]. Several limitations should be acknowledged. The cross-sectional design does not allow for the assessment of the longitudinal dynamics of IL-15 in relation to disease progression; IL-15 was analyzed as a single circulating biomarker without broader cytokine profiling; the small group sizes may have limited the statistical power to detect differences; the wide age range may have affected the reliability and clinical applicability of the results; urinary IL-15 concentrations were not normalized to urinary creatinine, which can affect the results by urine dilution; the study did not quantify cyst size, total cyst number, or total kidney volume (ht-TKV); and the logistic regression models showed wide confidence intervals, indicating statistical imprecision and substantial variability of the estimates. Although ROC analysis showed high AUC values for serum and urinary IL-15, these results were obtained in a small, single-center cohort and were not validated in an independent population. The present findings contribute additional insight into pediatric cystic kidney disease and highlight IL-15 as a potentially relevant component of this process. Clarifying its exact biological and clinical role will require further investigation in larger and longitudinal pediatric cohorts.

## 5. Conclusions

Elevated serum and urinary IL-15 concentrations were observed in children with cystic kidney disease compared with controls. IL-15 levels were not associated with current renal function and did not differ between cystic disease subtypes, suggesting that IL-15 upregulation occurs independently of measurable functional impairment. When interpreted alongside available pediatric and adult data, these findings indicate that IL-15 may reflect early inflammatory or epithelial signaling rather than established renal dysfunction. Given the limited sample size and lack of validation, the diagnostic utility of IL-15 should be considered exploratory and requires confirmation in larger, independent, and longitudinal pediatric cohorts. Further longitudinal studies are needed to clarify the clinical relevance of IL-15 in pediatric cystic kidney disorders.

## Figures and Tables

**Table 1:** Anthropometric, age, sex and blood pressure parameters in children with renal cystic disease and controls.

Parameter	Renal Cystic Disease (n = 47)	Control Group (n = 41)
Height (cm)	142.5 ± 26.3 (72.0–184.5)	136.4 ± 25.3 (82.0–197.0)
SDS for height	0.1 ± 1.2 (−2.9–3.8)	0.2 ± 1.1 (−1.5–3.0)
Body weight (kg)	42.7 ± 20.1 (8.8–81.0)	35.5 ± 19.3 (9.7–87.5)
SDS for body weight	1.1 ± 2.4 (−2.8–10.6)	0.1 ± 1.1 (−2.3–2.1)
BMI (kg/m^2^)	19.6 ± 4.3 (13.6–29.9)	17.6 ± 3.5 (12.4–26.5)
Age (years)	10.6 ± 4.4 (0.8–18)	9.3 ± 4.2 (2.0–17.5)
Female sex (n%)	25 (53.2%)	17 (41.5%)
Male sex (n%)	22 (46.8%)	24 (58.5%)
SDS for BMI	1.2 ± 2.2 (−2.0–6.8)	−0.1 ± 1.2 (−2.9–2.3)
* SYS (mmHg)	110.1 ± 11.4 (90.0–135.0)	111.8 ± 11.3 (85.0–134.0)
* DIA (mmHg)	64.1 ± 7.4 (50.0–84.0)	69.5 ± 11.6 (45.0–107.0)
* MAP (mmHg)	79.6 ± 7.9 (63.3–99.7)	79.3 ± 11.4 (59.3–115.7)

Data are presented as mean ± standard deviation (minimum–maximum). Sex is presented as n (%). SDS, standard deviation score; BMI, body mass index; SYS, systolic arterial pressure; DIA, diastolic arterial pressure; MAP, mean arterial pressure. * for n = 41.

**Table 2:** Comparison of serum and urinary IL-15 concentrations between cystic kidney disease subgroups.

Parameter	ADPKD (n = 23)	Simple Renal Cysts (n = 10)	Other Cystic Kidney Disease (n = 14)	*p*-Value
Serum IL-15 (pg/mL)	4.8 (4.5–5.1)	4.8 (4.5–5.1)	4.6 (4.3–5.0)	0.767
Urinary IL-15 (pg/mL)	4.1 (3.8–4.2)	4.1 (4.04–4.2)	4.1 (3.8–4.2)	0.701

Values are presented as median (Q1–Q3).

**Table 3:** Correlations between serum and urinary IL-15 concentrations and selected biochemical parameters in the study group.

Parameter	Serum IL-15 R	Serum IL-15 *p*-Value	Urinary IL-15 R	Urinary IL-15 *p*-Value
CRP	−0.040	0.805	0.002	0.989
eGFR	0.115	0.440	−0.021	0.889
Serum IL-15	-	-	0.634	<0.001
Urinary IL-15	0.634	<0.001	-	-

Data are presented as Spearman’s rank correlation coefficients (R) with corresponding *p*-values. Correlations were assessed between serum IL-15 and urinary IL-15 concentrations and selected biochemical parameters, including C-reactive protein (CRP) and the estimated glomerular filtration rate (eGFR). A *p*-value < 0.05 was considered statistically significant. IL-15, interleukin-15; CRP, C-reactive protein; eGFR, estimated glomerular filtration rate. N = 47.

**Table 4:** Univariable logistic regression analysis of renal cyst presence.

Variable	OR	95% CI	SD	*p*-Value
Serum IL-15	59.9	14.3–430.9	0.9	<0.001
Urinary IL-15	9.8	4.2–27.2	0.5	<0.001

Univariable logistic regression analysis was performed to evaluate the association between serum and urinary IL-15 concentrations and the presence of renal cysts. Results are presented as odds ratios (ORs) with standard deviations (SDs), 95% confidence intervals (CIs), and *p*-values. IL-15, interleukin-15; OR, odds ratio; SD, standard deviation; CI, confidence interval.

**Table 5:** Diagnostic performance of serum and urinary IL-15 for renal cyst presence.

Variable	Threshold	Specificity	Sensitivity	AUC
Serum IL-15	4.2	0.9	0.9	0.9
Urinary IL-15	3.7	0.9	0.8	0.9

ROC curve analysis was used to assess the diagnostic performance of IL-15. Thresholds were determined using the optimal cut-off point.

## Data Availability

The data presented in this study are available on request from the corresponding author. The data are not publicly available due to privacy issues.
